# Adolescents' perceptions about smokers in Karnataka, India

**DOI:** 10.1186/1471-2458-11-563

**Published:** 2011-07-14

**Authors:** Upendra M Bhojani, Maya A Elias, Devadasan N

**Affiliations:** 1 Institute of Public Health, 250, 2 C cross, 2 C main, Girinagar 1st phase, Bengaluru, Karnataka, India

## Abstract

**Background:**

Prevalence of tobacco use among adolescents in India is very high. Despite many epidemiological studies exploring tobacco use among youth, there is no published data on adolescents' perceptions about smokers in Indian society and its implications on tobacco control.

**Methods:**

A cross-sectional study was conducted using a stratified random sampling with probability proportional to school-type (government or private owned). Data was collected using a pretested, self-administered, anonymous questionnaire with a mix of close and open-ended questions from a sample of 1087 students. Chi-square test was used to measure associations. Qualitative data was analysed through inductive coding.

**Results:**

The response rate for the study was 82.5% and the sample population had a mean age of 16.9 years (SD = 1.9) with 57.8% male students. Majority of respondents (84.6%) reported negative perceptions about smokers while 20.4% of respondents reported positive perceptions. Female students reported significantly higher disapproval rate (negative perceptions) for smoking compared to male students (89.7% Vs 71.6% in case of male smoker; 81.2% Vs 67.3% in case of female smoker). Dominant themes defining perceptions about smokers included 'hatred/hostility/Intolerance', 'against family values/norms', 'not aware of tobacco harms' and 'under stress/emotional trauma'. Themes like 'culture', 'character' and 'power' specifically described negative social image of female smoker but projected a neutral or sometimes even a positive image of male smoker. There was a significant association between adolescents' positive perceptions of smokers and tobacco use by themselves as well as their close associates.

**Conclusions:**

Adolescents' stereotypes of smokers, especially female smokers are largely negative. We suggest that tobacco control interventions targeting adolescents should be gender specific, should also involve their peers, family and school personnel, and should go beyond providing knowledge on harmful effects of smoking to interventions that influence adolescents' social construct of smoking/smoker.

## Background

Prevalence of tobacco use among adolescents in India is quite high. The Global Youth Tobacco Survey (GYTS) in 2006 indicated that the national prevalence of current tobacco use among school-going adolescents (between 13 to 15 years of age) was 14.1% and had not changed significantly from the GYTS 2003 (16.9%) [1]. Despite the growing number of epidemiologic studies examining tobacco use among youth in India, there is paucity of information regarding how 'a smoker' is perceived by adolescents.

An ecological perspective on health related behaviour change theories emphasises multiple levels of influence i.e. individual, interpersonal and community levels, that affect behaviour change [2]. One common factor that operates at all these levels of influence is the psycho-social construction of the behaviour in question, whether in the forms of personal beliefs, opinions, subjective norms, or social norms. This suggests that perceptions about smokers (at the personal, interpersonal and community levels) constitute one of the factors that may influence adolescents to adopt or reject smoking behaviour.

Hence, understanding of perceived images of smokers by adolescents may help in understanding adolescents' behaviour regarding tobacco use and in framing effective tobacco control interventions. This is especially important in the Indian context given the increasing emphasis for school-based tobacco control interventions as reflected from the objectives of the recently launched National Tobacco Control Programme by Health Ministry [3].

Though GYTS in India collected data on adolescent's perceptions about smokers, these findings were not brought into the subsequent reports and dissemination materials to inform policy makers and programme managers [1,4,5]. This paper presents a component of the Bengaluru Youth Tobacco Study (BYTS) conducted by the Institute of Public Health, Bengaluru. The main objectives of the BYTS were to assess (1) tobacco use; (2) perceptions about tobacco use among adolescents; (3) reasons for use or non use of tobacco products; and (4) the extent to which selected tobacco control policies were being implemented. This paper focuses on the first and second objectives (i.e. adolescents' tobacco use and their perceptions about smokers and its correlates) and discusses the implications of the findings on tobacco control.

## Methods

### Research design

It was a cross sectional study carried out in educational institutions (11^th ^and 12^th ^grade students) in Bengaluru urban district of Karnataka state from July 2009 to January 2010.

### Setting and sampling

Bengaluru (formerly known as Bangalore) is the metropolitan capital of the Indian state of Karnataka with a population of more than 6.5 million people. As per records available for the year 2006-2007, Bengaluru urban district had 409 schools providing education for 11^th ^and 12^th ^grades [6].

An estimated tobacco use prevalence of 15.6% in a similar age population in Bengaluru city was used to arrive at the sample size of 575 (n=(1.96)^2 ^× 0.16 × 0.84⁄(0.03)^2^) considering 95% confidence level and +/- 0.01 precision [7]. For this study, randomisation was at the level of the school and the class and not at the level of individual students. In order to compensate for design defects and student absenteeism a further 50% was added to the estimated sample size. Assuming an average enrolment size of 50 students per class, the sampling frame required us to select 19 schools to achieve the target sample of 863 students.

A two-staged stratified random sampling method was used. In the first stage, a detailed list of all the schools providing education for 11^th ^and 12^th ^standards in the Bengaluru urban district was obtained from the state education department. Schools were stratified based on school ownership and were randomly selected based on probability proportional to school (ownership) type. In the second stage, two classes from each of the sampled schools were randomly selected. All the students present in the selected classes on the day of the survey were eligible to participate in the study.

### Data collection and management

Data was collected using a pretested, anonymous, self-administered questionnaire (in English as well as in Kannada, the local language in the state) with mostly close-ended questions and a few open-ended questions. A copy of the questionnaire can be found in the Additional File 1. After explaining the purpose of the survey and the voluntary and anonymous nature of participation in the survey, two of the authors (UMB and MAE) distributed the questionnaires to those students who agreed to participate in the survey. The researchers remained present throughout the survey to clarify any queries. The same researchers conducted the survey in all the sampled schools. Data entry was done by two trained data entry operators using EpiData 3.1 (2008). Random verification of 10% of data was done and internal validity checks were performed.

Researchers' institution did not require formal ethical approval for the research at the time of the study. In order to ensure ethical conduct of the research, various measures were taken by reseachers including (1) written permission from the education department to conduct the study; (2) prior written consent from the heads of the educational institutions; (3) verbal consent from participating students; and (4) anonymity of participating institutions and students as well as data security. Study findings were shared with the participating educational institutions in form of a factsheet. Researchers delivered education on harms of tobacco use and related aspects to participating students in form of an interactive lecture.

### Measures and analysis

Adolescent's perceptions about smoker were assessed by the following questions...

➢ When you see a man smoking, what do you think of him?

➢ When you see a woman smoking, what do you think of her?

To answer these questions, respondents were given multiple descriptors to choose from (i.e. Lacks confidence, stupid, loser, immoral, successful, intelligent, smart, sophisticated, macho (in case of male smoker) and bold (in case of female smoker)). Respondents had the choice to select as many descriptors as they felt relevant. Apart from these given descriptors, respondents were given a choice to express any other feelings that they have when they see a man or woman smoking as an open-ended response. These questions and descriptors were adopted from the validated GYTS core questionnaire used in Karnataka [4].

Ever tobacco user referred to a person who used any form of tobacco products even once during his/her lifetime. Current smoker referred to one who used any form of smoking tobacco products within 30 days preceding the survey. These were categorical variables with a yes/no answer.

On-Screen tobacco use referred to portrayal of use of any tobacco products by actors on television or in films. Adolescents' exposure to on-screen smoking (one year recall) as well as to cigarette advertisements (thirty days recall) was assessed by asking them to recall such exposure and choose one of the four response categories (i.e. sometimes, a lot of the times, never, not watched television/films). Adolescents' exposure to tobacco use by teachers and close friends was assessed by asking adolescents to select one of the four response categories indicating proportion of their teachers/friends using tobacco (i.e. all of them, some of them, most of them, none of them). Tobacco use in family was assessed by asking students to report tobacco use by their parents as well as siblings.

EpiInfo™8.5.1 (2008) was used to do univariate and bivariate analysis. Correlates of adolescents' perceptions about smokers were analysed using chi-square tests. Responses generated through open-ended response categories were post coded through inductive coding and were described using overarching themes.

## Results

In total, 19 schools having fair geographic distribution across the district participated in the survey. Due to higher enrolment and relatively low absenteeism the total sample size achieved was 1087 students. A response rate of 100% was obtained at the level of the schools and students present on the day of the survey. Accounting for those students who remained absent on the day of the survey, the overall response rate for the survey was 82.5%. The item non-response varied from 19.58% to 20.49%. Sample population had a mean age of 16.9 years (SD = 1.9) with 57.8% male students.

### Tobacco use by students

We found that 15.1% of students had used tobacco at least once in their life with more males (21.7%) using tobacco compared to females (5.3%) (OR = 5, 95%CI=(3.3, 8.2)). We found that 7.2% of students were current users of tobacco with similar trend of higher prevalence among male compared to female students (11.2% Vs 1.5%). Tobacco use among students from government and private managed schools was 16.7% and 15% respectively. Tobacco use was significantly higher among students having friends using tobacco (22.2%) compared to students having friends who don't use tobacco (2.1%) (OR = 13.4, 95%CI=(6.8, 29.6)). We found significant positive association between tobacco use by students and tobacco use by their family members (OR = 2.1, 95%CI=(1.4, 3.1)). Among students who were exposed to school personnel using tobacco, tobacco use was significantly higher (27.6%) compared to their counterparts (9.1%) (OR = 3.8, 95%CI=(2.7, 5.4)).

### Adolescents' perceptions about male and female smokers

As shown in Table 1, majority of respondents (84.6%) reported having negative perceptions about smokers. Only 20.4% of respondents reported positive perceptions about smokers. Negative perceptions included a smoker being stupid, loser, immoral, and someone who lacks confidence in a descending order of prominence in case of both, male and female smokers. On an average more students had negative perceptions about male smoker compared to female smoker. However, in terms of morality, comparatively higher proportion of students perceived female smoking as immoral compared to male smoking. Though proportion of students having positive perceptions about female smoker was higher compared to male smoker, it was only due to significant higher proportion of students (13.7% male and 14.1% female students) who perceived female smoker as 'bold' compared to very few students who perceived male smokers as 'macho' (2.2% male and 1.3% female students). In all other aspects, proportion of students perceiving male smokers positively was higher compared to female smokers.

**Table 1:** Adolescents' perceptions about smokers, Bengaluru, India.

	Perceptions about a male smoker (n = 1048)*		Perceptions about a female smoker (n = 1060)*		Overall (n = 1060)*
	Male students (n = 603)	Female students (n = 445)	Male students (n = 613)	Female student (n = 447)	
**Negative perceptions (%)**	**71.6**	**89.7**	**67.3**	**81.2**	**84.6**
*Stupid *	28 (24.5, 31.8)	55.1 (50.3, 59.7)	34.9 (31.2, 38.9)	50.6 (45.8, 55.3)	
*Loser*	32.3 (28.6, 36.3)	39.8 (35.2, 44.5)	18.6 (15.6, 22)	25.7 (21.8, 30.1)	
*Immoral*	14.8 (12.1, 17.9)	16 (12.7, 19.8)	16.3 (13.5, 19.5)	23.5 (19.7, 27.8)	
*Lacks confidence*	10 (7.7, 12.7)	9 (6.6, 12.1)	8.8 (6.7, 11.4)	8.1 (5.8, 11.1)	
**Positive perceptions (%)**	**14.9**	**7.2**	**20.4**	**17.7**	**20.4**
*Successful*	2.8 (1.7, 4.6)	0.9 (0.3, 2.4)	2 (1.1, 3.5)	0.7 (0.2, 2.1)	
*Intelligent*	3.6 (2.4, 5.6)	2.2 (1.1, 4.2)	1 (0.4, 2.2)	0.9 (0.3, 2.4)	
*Smart*	3.5 (2.2, 5.4)	0.2 (0, 1.4)	2 (1.1, 3.5)	0.4 (0.1, 1.8)	
*Sophisticated*	4.3 (2.9, 6.3)	3.1 (1.8, 5.3)	2.6 (1.6, 4.3)	2.2 (1.1, 4.2)	
*Macho (male smoker)*,	2.2 (1.2, 3.6)	1.3 (0.5, 3.1)	-	-	-
*Bold (female smoker)*	-	-	13.7 (11.1, 16.7)	14.1 (11.1, 17.7)	
**Others (%)**	**21.6**	**13.5**	**19.6**	**13.2**	**23.4**

*Total percentage (proportion of students having 'positive perceptions', 'negative perceptions' and 'others') exceed that of 100% due to multiple responses. Number of respondents in the two columns differs because of difference in response rate for these variables (96.4% and 97.5% respectively)

### 'Other' perceptions about male and female smokers

Interpretation of responses under 'others' category provides interesting insights regarding social, cultural, and political perceptions about smokers. In regard to perceptions of male smokers, a total of 152 respondents (97 males and 54 females) opted for the 'others' category. Of these, 72 male respondents (74.2%) and 41 female respondents (75.9%) provided detailed responses. In regard to perceptions about female smokers, a total of 145 respondents (91 males and 54 females) chose the 'others' category. Of these, 66 male respondents (72.5%) and 41 female respondents (75.9%) provided detailed responses. Table 2 depicts overarching themes reflecting adolescents' perceptions about smokers along with use of quotes explaining the themes.

**Table 2:** Themes defining adolescents' perceptions about smokers.

Dominant themes common in defining perceptions about smokers
**Under stress/emotional trauma/love failures** ["He(MS*) would be in some tension", "He(MS) would be in some feeling, which would pain his heart", "Devadas ^± ^"], ["she(FS^^^) is under tension/depression"] - male students ["He(MS) is depressed and needs help", "(MS smokes) Because of tension", "Home problem or love failure", "His(MS's) personal feeling like disappointment of love"], ["(FS smokes) to forget sadness in the life", "She(FS) may have some problem", "My feeling is that they(FSs) use whenever they are depressed"] - female students **Against family values/norm** **[**"He(MS) is left free by their parents", "How unlucky are his(MS's) family members"], ["She(FS) has not grown up properly", "They(FSs) are not fit to be parents"] - male students ["He(MS) doesn't have care for his children who might learn to use tobacco from him], ["(FS) has a very bad family background"] - female students **Not aware of harms of tobacco** **[**"(MS) lacks knowledge", "...not conscious of life and don't know the truth"], ["She(FS) is illiterate and has no idea of its dangerous consequences"] - male students ["He(MS) is not having any knowledge about tobacco", "(MS is) Unaware of consequences"], ["(FS is) unaware of consequences", "(FS) lacks knowledge and is nonsense"] - female students **Hatred/hostility/intolerance** ["I feel like slapping him(MS) when he(MS) smoke in front of me" "I get irritated and feel like kicking him(MS)"], ["I would like to slap her(FS) or kill her(FS)", "She(FS) is not worth being alive"] - male students ["I hate them(MSs)", "I like to scold them(MSs)", "I like to slap him(MS) in the way"], ["I will like to kill her(FS)", I will like to slap her(FS)"] - female students

**Dominant themes specifically defining perceptions about female smokers**

**Antithetical to Indian culture** ["She is spoiling the social culture and is a great insult to women society", "She is not an Indian cultured women"] - male student ["They are insulting our culture", "She is not fit as a woman", "They are foreigners. They are from foreign land"] - female student **Bad character** ["I feel she is a prostitute"] - male students ["I think she is a very bad women", "Being a girl how can she even lose her standards?"] - female students **Gender power** ["She may think that a man can smoke then why can't a woman do that, because men and women should be equal", "She thinks we (women) are better than men in smoking"] - male students ["She thinks that she is compared to men", "Crazy! Don't ever try to compete with men what so ever it matters"] - female students

*Male smoker ^^^Female smoker ^± ^A male character of a legendary Bengali novel that suffered lifelong pain and frustration for not getting his childhood sweetheart whom he loved intensely and selflessly.

These themes largely reflected negative image of a smoker especially female smoker. Themes like 'hatred/hostility/Intolerance', 'against family values/norms', 'not aware of tobacco harms' and 'under stress/emotional trauma' commonly emerged from responses of male and female students for smokers. Themes like 'culture', 'character' and 'power' described negative social image of female smoker (being antithetical to Indian culture, having bad character and trying to be equal to men against expected norms) but projected a neutral or sometimes even a positive image of male smoker (being superior/powerful gender). Very limited positive perceptions about smokers were in regard to male smokers by very few male as well as female students e. g. "He (male smoker) looks cool" - female student, "it (smoking) gives sexy or love feeling" - male student. Some male students perceived that among females, smoking adds to sex appeal and sophistication e.g. "She (female smoker) is a hot woman", "They (female smokers) are mostly sophisticated women because they think they look smart by doing that (smoking)", and "She (female smoker) is quite sophisticated".

### Correlates of adolescents' perceptions about smokers

It is beyond the scope of this study to explain complex interplay of many factors that may shape adolescents' perceptions about smokers in society. However we use some of the relevant factors that we have measured in our study and explore their association with adolescents' perceptions about smokers.

#### Perceptions of smokers based on gender of students

**Figure 1:**
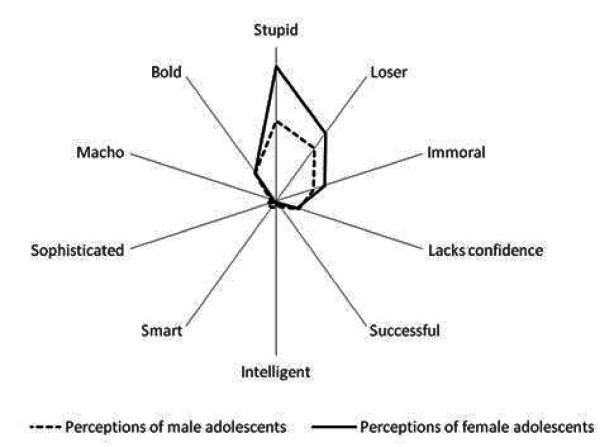
**Adolescents perceptions of smokers based on sex of adolescents**.

On an average, more male students, represented by the dotted line in Figure 1, reported positive perceptions about smokers compared to female students. This difference was found both in regard to perceptions about male as well as female smokers (14.9% Vs 7.2% in regard to male smokers; and 20.4% Vs 17.7% in regard to female smokers). This difference in male and female students' positive perceptions of smokers was statistically significant in case of male smoker but was not significant in case of female smoker. In other words, male students were more approving of male smoking compared to female students.

An unexpected finding was the perceived association of smoking behaviour with the morality of smoker. Not only were female smokers strongly perceived as 'immoral' compared to male smokers (39.8% Vs 30.7%) but there was a gender bias in that more female respondents associated female smokers with immorality compared to male respondents (23.5% Vs 16.3%) indicating internalisation of societal mores among female respondents.

#### Perceptions of smokers based on tobacco use by students

**Figure 2:**
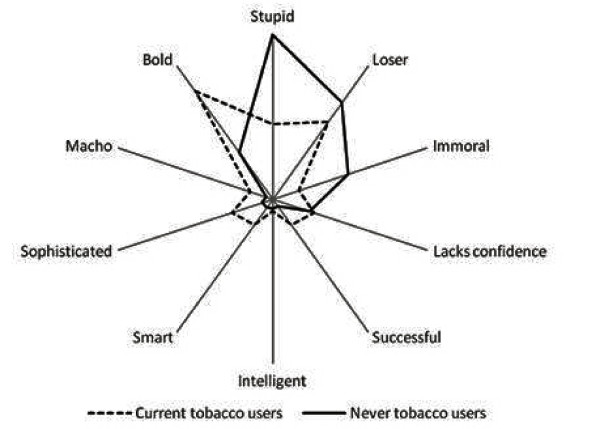
**Perceptions of male adolescents about smokers based on tobacco use status of adolescents**.

Apart from the gender, tobacco use status of the students appeared to be another significant factor associated with their perceptions about smokers. As shown in Table 3, adolescents who used tobacco (ever tobacco users as well as current smokers) were much more likely to have positive perceptions about a smoker. Figure 2 further provides comparison of male never tobacco users' as well as male current tobacco users' perceptions about smokers. Comparatively, male current tobacco users had more positive perceptions of smokers compared to male never tobacco users. This difference was found both in regard to perceptions about male as well as female smokers (39.1% Vs 13.2% in case of a male smoker; and 45% Vs 17.7% in case of a female smoker). These differences in perceptions of smokers between male current tobacco users and male never tobacco users were statistically significant. Similar data on female respondents is not presented here due to very limited number of female current tobacco users.

**Table 3:** Correlates of adolescents' perceptions about smokers

Correlates	Referent category for Odds Ratio (highlighted in bold)	Positive perceptions^* ^(%)	Odds Ratio (95% CI)	p value
**Gender**	**Male**	11.2	2.89	< 0.05
	Female	4.2	(1.59, 5.24)	
**Tobacco use status**	**Ever tobacco users**	24.3	5.72	< 0.05
	Never tobacco users	5.3	(3.34, 9.82)	
	**Current smokers**	42.2	12.99	< 0.05
	Never tobacco users	5.3	(6.16, 26.81)^^^	
**Exposure to others using tobacco**	**Having smoker friends**	12.8	4.65	< 0.05
	Having smoke-free friends	3.1	(2.44, 8.84)	
	**Having friends who used tobacco in any form (smoking and/or chewing)**	12.4	4.64	< 0.05
	Having tobacco-free friends	3.0	(2.39, 9.01)	
	**Having one or more family members smoking tobacco**	9.7	1.56	0.11
	Having smoke-free family	6.4	(0.89, 2.71)	
	**Having one or more family members using tobacco**	10.1	1.71	0.06
	Having tobacco-free family	6.2	(0.96, 3.06)	
	**Exposure to teachers using tobacco**	13.4	2.42	< 0.05
	Not exposed to teachers using tobacco	6.0	(1.44, 4.07)	
	**Exposure to on-screen tobacco use**	7.6	0.60	0.22
	No exposure to on-screen tobacco use	12.1	(0.25, 1.65)^^^	
**Awareness on**	**Aware of harms of smoking**	7.5	0.62	0.23
**smoking harms**	Not aware of smoking harms	11.6	(0.28, 1.37)	
	**Awareness of harms of passive smoking**	7.4	0.81	0.41
	Not aware of harms of passive smoking	8.9	(0.31, 2.75)^^^	
**Exposure to Cigarette**	**Exposure to cigarettes advertisements**	8.7	1.23	0.43
**advertisements**	No exposure to cigarette advertisements	7.2	(0.73, 2.06)	
**School type**	**Government managed**	6.6	0.79	0.59
	Private managed	8.2	(0.30, 1.79)	

*This data represents proportion of students who had only positive or negative perceptions of smokers and exclude those students who reported ambivalent perception of smokers. ^^^Data using Fisher exact test as one of the table value was less than five.

### Other correlates

Tobacco use by friends and teachers were found to be significantly associated with positive perceptions of smoking by adolescents. Other factors that showed positive association but failed to reach statistical significance included exposure of students to cigarette advertisements, and tobacco use by their family member/s. Factors that were negatively associated with student's positive perceptions about smoking but did not achieve statistical significance included awareness about the harmful effects of smoking, and studying in a government-managed school. Though not significant, it was counterintuitive to note that there was a negative association between exposure to on-screen smoking and positive perceptions of smokers.

## Discussion

Through this study, we make a few important observations. First of all, the quantitative and qualitative data presented in the paper provides myriad social, cultural, and political connotations of smoking as perceived by adolescents in the Indian society. This social construct of the smoker may be one of the important factors influencing adolescents' decision regarding uptake of smoking. This study found that adolescents had predominantly negative perceptions about both male and female smokers. There are no similar Indian studies with the same age-group to compare our results. However our analysis of the data from GYTS Karnataka (2003) that was carried out among school-going students in the age-group 13 to 15 years of age using a random, probability proportional to enrolment size sample also revealed predominantly negative perceptions of smokers with 95.1% adolescents indicating negative perceptions of male smokers and 74.9% of adolescents with negative perceptions of female smokers (Data not presented in this paper).

Perceived association of smoking as a mechanism to relieve stress, look fashionable, sophisticated or even romantic or sexy as reported by adolescents point to their vulnerability for taking up tobacco use. This study found a significant association between tobacco use by adolescents and their positive perceptions of an adult smoker.

On the other hand, negative perceptions of adult smoker may have a protective influence for adolescents for not using tobacco given the significant positive association between these two variables found in this study. Studies from United States of America suggest positive impact of social unacceptability of smoking on reducing tobacco use and points towards perceived smoking related stigma as a possible mechanism making smokers more likely to quit [8,9]. Our study points to the need for prospective studies to understand causal relationships between adolescents' tobacco use and their social construct of smokers.

Our study found a significant gender bias in that female smokers generated extremely negative responses on moral ground from students compared to male smokers and that female students were significantly more disapproving of smoking behaviour compared to male students. Qualitative findings from the study complemented this impression of the gender bias in terms of social unacceptability of female smokers where themes like 'culture', 'character' and 'power' described negative social image of female smoker but projected a neutral or sometimes even a positive image of male smoker (being superior/powerful gender). This scenario appears to fit into the prevailing social construct of gender and tobacco use in Asian countries where tobacco use is often linked to expression of masculinity while having strong social sanction against female smoking [10-14].

There are limitations of this study. The sample of the study was drawn from school-going adolescents in the age group of 15 to 18 years from the Bengaluru urban district of Karnataka. Given the metropolitan nature of Bengaluru, the study findings may only be generalised to all school-going adolescents in Bengaluru and not other urban areas in the state. Also the study relied on self-reported observations and did not use any other mechanism to validate the self-report.

Furthermore, perceptions of smokers by adolescents are shaped by complex interactions of many factors extending beyond the intimate world of adolescents to the larger socio-psycho-cultural-political environment. Our study captures only some of the factors related to adolescents and their close associates and hence cannot explain all the reasons for the given perceptions of smokers. Methodologically, no attempt to establish causality has been made.

## Conclusions

Given the limitations, the major findings of this study have important implications for tobacco control among youth. We believe that most tobacco control interventions aimed at schools-going adolescents in India are limited to provision of knowledge on harms of smoking or other tobacco forms. Findings from this study suggest that school based interventions aimed to reduce tobacco use among adolescents need to also include strategies to address perceptions of adolescents about smoking/smokers in addition to information on harmful effects of smoking and other policy level changes. Furthermore, as demonstrated by this study, such interventions should be gender specific to consider gender based differences in psycho-social construct of smoking/smokers.

In our study, the extent and intensity of negative perceptions of adult smokers (especially so in case of female smokers) points to the possible stigmatisation of smokers in Indian society. Smokers' stigmatisation is clearly beyond the scope of this study, but given the paucity of studies on this aspect in India, this study points to the need to understand smoking related stigmatisation in India.

Finally it is important to consider that positive perceptions of smokers by adolescents were significantly associated with tobacco use by adolescents and their close associates. In India, Global school Personnel Survey (GSPS 2006) indicated current tobacco use prevalence of 12.8% for cigarette smoking and 23.7% for other tobacco products among school personnel [1]. Hence the tobacco control efforts directed towards peers, family members and school personnel and their involvement is crucial to reduce tobacco use among adolescents.

## Competing interests

We declare that we have no competing interests.

## Authors' contributions

UMB and DN were involved in designing the study. UMB and MAE did data collection. UMB analysed the data and wrote the first draft manuscript. DN and MAE reviewed and revised the manuscript. All the three authors approved the final version submitted to the journal.

## Funding

This study was carried out under ISEC-SRTT Visiting Fellowship programme offered by the Institute for Social and Economic Change, Bengaluru, India.

## Pre-publication history

The pre-publication history for this paper can be accessed here:

http://www.biomedcentral.com/1471-2458/11/563/prepub

## Supplementary Material

Additional file 1 **Questionnaire**. Detailed questionnaire (English language) used in the study. Click here for file
