# Supplementary material for: Adolescents' perceptions about smokers in Karnataka, India
Source: BMC Public Health. 2011 Jul 14;11:563. doi: 10.1186/1471-2458-11-563 (PMC3150267; doi:10.1186/1471-2458-11-563)
Supplement: Additional file 1 — Questionnaire. Detailed questionnaire (English language) used in the study. [file 1471-2458-11-563-S1.PDF]

**'Confidential' - For research purpose only**

# Questionnaire to assess tobacco use and related factors among the pre-university college students

## Instructions

- This is a questionnaire to be filled by pre-university college students. Objective of this questionnaire is to know about tobacco use and related factors among Pre-university college students in Bangalore city.

- Students are requested to respond to all the questions. It may take around 30 minutes to fill this questionnaire. Please provide honest and correct answers to all the questions, as these are meant purely for research and welfare of young generation.

- Please note that you will not be asked to provide your name or student number or even the name of your PUC. Hence, your responses will remain completely ANONYMOUS. We will collect and protect the filled questionnaires and will not share it with the school authority or anyone else other than the members of the research team.

- You have been provided an Answer Sheet. Do not tick or write anything in the questionnaire. Always use Answer Sheet to provide your responses.

- In case of multiple-choice questions, please select the appropriate answer by making a circle with pencil or pen. Some questions allow you to select just one answer while others allow you to select as many answers as applicable. Kindly refer to the instruction given in the bracket after each question to decide the number of answers you are allowed to select.

  For example…

  1. Do any of your family members smoke tobacco? (Select as many responses as applicable)
     a. Father
     b. Mother
     c. Brother
     d. Sister
     e. Others in the family (please specify) ________
     f. None of them smoke tobacco
     g. I don't know

| | | | | | | | |
|---|---|---|---|---|---|---|---|
| 1 | a | b | c | d | f | g | |
| | e grandfather | | | | | | |

- Some questions require you to put your response in a box. In such cases, always use numbers as your response and fill the box with appropriate number. Follow any instructions given in the bracket beside the question.
  For example…

  2. How old were you when you first tried or experimented with any form of tobacco? (Select only one response)
     a. I have never tried or experimented with tobacco
     b. I was ☐ years old when I first tried or experimented with tobacco

| 2 | a | b (circled) 9 Years | |
|---|---|---|---|

- A very few questions may require you to describe your response in writing. They provide a blank space for you to write a response. Please try to describe your main belief/argument within the given space
  For example…

  3. What were the reasons you started smoking? (Select only one response)
     a. I have never smoked tobacco
     b. I started smoking because of the following reason/s:
     ____________________________________________

| 3 | a |
|---|---|
| | b (circled) My friends were smoking. They often insisted me to smoke with them. They used to offer me cigarettes and one day I tried. Then after I also started using it regularly. |

- In case of any doubt or query please raise your hand and the researcher will attend you to resolve your doubt or query

- We highly appreciate the time and efforts that you will put in filling out this questionnaire. We commend your contribution towards improving understanding of tobacco use and related factors

## Personal Details

1. Age (number of completed years): [ ] years

2. Gender (Select only one response): a. Male b. Female

3. Standard of study (Select only one response): a. 1st PU b. 2nd PU

4. Study medium (Select only one response): a. Kannada b. English

5. Study stream (Select only one response):
   a. Science b. Arts/Humanities c. Commerce

6. Percentage of marks obtained in 10th grade: [ ] %

7. Religion (Select only one response):
   a. Hindu b. Muslim c. Shikh d. Christian e. Jain
   f. Buddhiest g. Others (please specify): _______________

8. Caste/tribe:
   a. Scheduled caste (SC) b. Scheduled tribe (ST)
   c. Other backward class (OBC) d. Others
   e. Don’t know

9. Current residence area name (e.g. Jayanagar 7th block): _______________

10. Current residence area PIN (e.g. 560078): [ | | | | | ]

## Tobacco Use

11. Have you ever tried or experimented with any form of tobacco, even once or twice? (e.g. cigarette, bidi, pan with tobacco, gutka, khaini, pan-masala, tobacco-lime mixture etc. Select only one response)
    a. Yes b. No

**If your answer is b. then please directly proceed to the question no.31**

12. How old were you when you first tried or experimented with any form of tobacco? [ ] years

13. Which form of tobacco you used when you first tried tobacco? (Select only one response)

    a. I chewed tobacco (please specify the type of chewing tobacco i.e. gutka, khaini, pan with tobacco, raw tobacco, pan-masala etc.) ______________________________________________
    b. I smoked (please specify the type of smoking tobacco i.e. cigarette, bidi, etc.) ________________________________________
    c. I applied tobacco (please specify the type of tobacco application i.e. snuff, tooth paste containing tobacco, tobacco tooth powder etc.)____________________________________________

14. Did you shift from the type of tobacco you used first time to some other type of tobacco over the time? (Select only one response)
    a. I started chewing tobacco first and over the time I shifted to smoking tobacco
    b. I started chewing tobacco first and over the time I shifted to tobacco application
    c. I started with smoking first and over the time I shifted to chewing tobacco
    d. I started with smoking first and over the time I shifted to tobacco application
    e. I started applying tobacco first and over the time I shifted to chewing tobacco
    f. I started applying tobacco first and over the time I shifted to smoking
    g. I never shifted to other type of tobacco. I always used the same type of tobacco that I started first.
    h. I followed some other pattern (please specify) ______________ ________________________________________________
    i. Over the time I have used various forms of tobacco without following any particular pattern or sequence

15. Did you start tobacco use in last one year? (Select only one response)
    a. Yes, I started tobacco use in last one year
    b. No, I started tobacco use before one year

16. During the past 30 days (one month) did you use tobacco in any form? (Select as many response as applicable)
    a. I did not use tobacco in any form in past 30 days
    b. I smoked tobacco c. I chewed tobacco

d. I applied tobacco

17. During the past 7 days (one week) did you smoke tobacco in any form? (Select as many response as applicable)
    a. I did not smoke tobacco in any form in past 7 days
    b. I smoked cigarettes c. I smoked bidis
    d. I smoked some other type of tobacco (please specify) ____________

18. During the past 7 days (one week) did you chew tobacco in any form? (Select as many responses as applicable)
    a. I did not chew tobacco in any form
    b. I chewed pan with tobacco c. I chewed khaini
    d. I chewed pan-masala with tobacco.
    e. I chewed tobacco-lime mixture without supari
    f. I chewed tobacco-lime mixture with supari
    g. I chewed some other type of tobacco (please specify)____________

19. During the past 7 days (one week) did you apply tobacco in any form? (Select as many responses as applicable)
    a. I did not apply tobacco in any form
    b. I used a tooth paste containing tobacco (e.g. Ipco, Dentoback)
    c. I used a tooth powder containing tobacco (e.g. Lal Dantmanjan)
    d. I used snuff or bajjar or tapkir
    e. I applied tobacco directly to my teeth or gums
    f. I used misri or gudakhu
    g. I applied some other type of tobacco (please specify) _______

**If your answer to question no. 17, 18 and 19 is a. then please directly proceed to question no.31**

20. During the past 7 days (one week), how many days did you smoke tobacco in any form? (If you did not smoke tobacco in the past 7 days, put 0 in the box): ☐ days

21. During the past 7 days (one week), how many days did you chew tobacco in any form? (If you did not chew tobacco in the past 7 days, put 0 in the box): ☐ days

22. During the past 7 days (one week), how many days did you apply tobacco in any form? (If you did not apply tobacco in the past 30 days, put 0 in the box): ☐ days

23. During the past 7 days (one week), on the days you smoked, how many cigarettes did you usually smoke? ☐ Cigarettes

24. During the past 7 days (one week), on the days you smoked, how many bidis did you usually smoke? ☐ Bidis

25. During the past 7 days (one week), on the days you chewed tobacco, how many packets of gutka/pan-masala did you usually chew? ☐ packets

26. During the past 7 days (one week), on the days you chewed tobacco, how many pans (with tobacco)/tobacco-lime mixtures did you usually chew? ☐

27. How soon after you wake up do you use your first tobacco? (Select only one response)
    a. I have stopped using tobacco
    b. Within the first 5 minutes
    c. More than 5 minutes after waking but within 30 minutes
    d. More than 30 minutes but within the first hour (60 minutes)
    e. More than an hour but before noon
    f. In the afternoon
    g. In the evening

28. Do you use tobacco more during the first 2 hours than during the rest of the day? (Select only one response)
    a. I stopped using tobacco    b. Yes    c. No

29. During the past 7 days (one week), what brand of tobacco products did you commonly use? (Please also indicate if it was 'mild', 'ultra mild', 'Regular' 'long', 'mini', 'Silk cut' etc. Select as many responses as applicable)
    a. Brand name of cigarettes I mainly used in past 7 days is/are: ______________________________
    b. Brand name of bidis I mainly used in past 7 days is/are: ______________________________
    c. Brand name of gutka/khaini/pan-masala I mainly used in past 7 days is/are: ______________________________

## Expenditure on tobacco

30. During the past 7 days (one week), how much of total money do you think you spent on various tobacco products? (Select as many responses as applicable)
    a. I spent about Rs. ☐ on cigarettes in the past 7 days
    b. I spent about Rs. ☐ on bidis in the past 7 days
    c. I spent about Rs. ☐ on chewing tobacco in the past 7 days

31. In a usual week (7 days), how much of pocket money do you get (including money that you earn and keep with you)? (Select only one response)
    a. I do not get any pocket money (or do not have any income etc.)
    b. I get about Rs. ☐ as a pocket money in a usual week

## Exposure to others using tobacco

32. Do any of your family members smoke tobacco? (Select as many responses as applicable)
    a. Father b. Mother c. Brother
    d. Sister e. Others in the family (please specify)_______
    f. None of them smoke tobacco g. I don't know

33. Do any of your family members chew tobacco? (Select as many responses as applicable)
    a. Father b. Mother c. Brother
    d. Sister e. Others in family (please specify)_____________
    f. None of them g. I don't know

34. Do any of your family members apply tobacco? (Select as many responses as applicable)
    a. Father b. Mother c. Brother
    d. Sister e. Others in family (please specify)_____________
    f. None of them g. I don't know

35. During the past 7 days (one week), on how many days have people used tobacco in your presence, in places other than in your home? (If they did not use tobacco on any days, put 0 in the box): ☐ days

36. Do any of your closest friends smoke? (Select only one response)
    a. None of them b. Some of them
    c. Most of them d. All of them

37. Do any of your closest friends chew tobacco? (Select only one response)
    a. None of them b. Some of them
    c. Most of them d. All of them

38. Do any of the teachers at your College use tobacco in any form? (Select only one response)
    a. None of them b. Some of them
    c. Most of them d. All of them

39. Do any of the non-teaching staff (security, peons, clerks, driver etc.) at your College use tobacco in any form? (Select only one response)
    a. None of them b. Some of them
    c. Most of them d. All of them

**Benefits and harms of tobacco**

40. Do you think chewing tobacco helps in some ways? (Select only one response)
    a. Yes, chewing tobacco is helpful in some ways (please specify in what ways it helps): ____________________________________________
    ______________________________________________________
    b. No
    c. I am not sure

41. Do you think smoking helps in some ways? (Select only one response)
    a. Yes, smoking is helpful in some ways (please specify in what ways it helps): ____________________________________________
    ______________________________________________________
    b. No
    c. I am not sure

42. Do you think applying tobacco helps in some ways? (Select only one response)
    a. Yes, applying tobacco is helpful in some ways (please specify how it helps): ____________________________________________
    ______________________________________________________
    b. No
    c. I am not sure

43. Do you think smoking is harmful to your health? (Select only one response)
    a. Definitely not b. Probably not
    c. Definitely yes d. Probably yes
    e. I don’t know

44. Do you think chewing tobacco is harmful to your health? (Select only one response)
    a. Definitely not b. Probably not
    c. Definitely yes d. Probably yes
    e. I don't know

45. Do you think applying tobacco is harmful to your health? (Select only one response)
    a. Definitely not b. Probably not
    c. Definitely yes d. Probably yes
    e. I don't know

46. Do you think the smoke from other people's cigarettes or bidis is harmful to you? (Select only one response)
    a. Definitely not b. Probably not
    c. Definitely yes d. Probably yes
    e. I don't know

**Reasons for use/non use of tobacco products**

47. What are the reasons you tried OR never tried tobacco use? (Select as many responses as applicable)
    a. I never tried tobacco because of the following reason/s:
    ______________________________________________
    ______________________________________________
    ______________________________________________
    ______________________________________________
    b. I tried smoking because of the following reason/s:
    ______________________________________________
    ______________________________________________
    ______________________________________________
    ______________________________________________
    c. I tried chewing tobacco because of the following reason/s:
    ______________________________________________
    ______________________________________________
    ______________________________________________
    ______________________________________________

48. Have you tried any of the following substances? (Select as many responses as applicable)

a. Alcohol b. Marijuana (cannabis)
c. Other illicit substances please specify ____ d. None of these

49. Before trying or experimenting with tobacco, did you use any non-tobacco products regularly? (Select as many responses as applicable)
    a. I have never tried or experimented with tobacco
    b. I did not use any non-tobacco products regularly before trying with tobacco
    c. I used pan
    d. I used supari
    e. I used pan-masala (without tobacco)
    f. I used some other non-tobacco products regularly (please specify)___________________________________

**If your responses include a. please directly proceed to the question no.51**

50. Which of the following factors influenced you to take-up or to continue tobacco use? (Select as many responses as applicable)
    a. Friend/s b. Family members c. Relatives
    d. Movie stars e. Television stars f. Teachers
    g. People using tobacco at shopping malls
    h. Tobacco advertisements on Television
    i. Tobacco advertisements on bill boards/hoardings
    j. Tobacco advertisements in print media (magazines, news papers etc.)
    k. Other factors (please specify)___________________________

## Attitudes towards tobacco use

51. Do you think you will take up tobacco use in future? (Select only one response)
    a. I am currently using tobacco
    b. Yes
    c. No
    d. I am not sure

52. When you see a man smoking, what do you think of him? (Select as many responses as applicable to you)
    a. Lacks confidence b. Stupid c. Loser
    d. Successful e. Intelligent (brainy) f. Smart
    g. Sophisticated (cool) h. Macho (Manly OR masculine)
    i. Immoral j. Some other feelings (please specify) __

53. When you see a woman smoking, what do you think of her? (Select as many responses as applicable to you)
    a. Lacks confidence    b. Stupid    c. Loser
    d. Successful    e. Intelligent (brainy)    f. Smart
    g. Sophisticated (cool)    h. Bold    i. Immoral
    j. Some other feelings (please specify) ____________________

54. Do you think smoking tobacco makes boys look more or less attractive? (Select only one response)
    a. More attractive    b. Less attractive
    c. Does not make any difference    d. I am not sure

55. Do you think chewing tobacco makes boys look more or less attractive? (Select only one response)
    a. More attractive    b. Less attractive
    c. Does not make any difference    d. I am not sure

56. Do you think smoking tobacco makes girls look more or less attractive? (Select only one response)
    a. More attractive    b. Less attractive
    c. Does not make any difference    d. I am not sure

57. Do you think chewing tobacco makes girls look more or less attractive? (Select only one response)
    a. More attractive    b. Less attractive
    c. Does not make any difference    d. I am not sure

58. Do you think boys who smoke or chew tobacco have more friends or fewer friends as compared to boys who do not use tobacco? (Select only one response)
    a. More friends    b. Fewer friends
    c. Does not make any difference    d. I am not sure

59. Do you think girls who smoke or chew tobacco have more or fewer friends as compared to girls who do not use tobacco? (Select only one response)
    a. More friends    b. Fewer friends
    c. Does not make any difference    d. I am not sure

60. If one of your best friends offered you a cigarette, or bidi or a chewing tobacco, would you use it? (Select only one response)

a. Definitely not
b. Probably not
c. Definitely yes
d. Probably yes

61. Do you feel like using tobacco when you see your family members using tobacco? (Select only one response)
    a. Yes
    b. No

62. Do you feel like using tobacco when you see actors/stars using tobacco on TV or in movies? (Select only one response)
    a. Yes
    b. No

63. Please select one of the following opinions or provide your own opinion regarding whether people should be allowed to smoke or not.
    a. People should not be allowed to smoke even if they want to smoke because smoking harms their own health
    b. People should be allowed to smoke if they want to smoke as far as they smoke in a way that their smoking doesn't harm others.
    c. People should be allowed to smoke irrespective of harm caused by smoking to themselves or to others around them
    d. Your own opinion on whether people should be allowed to smoke or not and why.

    ______________________________________________
    ______________________________________________

**If you have never tried any form of tobacco, please directly proceed to question no.68**

**Tobacco quitting attitudes and practice**

64. Have you ever tried to stop smoking or chewing tobacco? Please provide reasons for doing so. (Select only one response)
    a. Yes, I have already stopped it because ____________________
    ______________________________________________
    ______________________________________________
    b. Yes, I have tried to stop it but I was not successful because_______
    ______________________________________________
    ______________________________________________
    c. No, I have never tried to stop smoking or chewing tobacco because__
    ______________________________________________
    ______________________________________________

65. Are you considering quitting tobacco use? (Select only one response)
    a. Yes  b. No  c. I have stopped using tobacco

66. Do you find it difficult to avoid tobacco use in places where it is generally not allowed (e.g. school classes, library, hospitals, church, temples, etc. Select only one response)?
    a. I have stopped using tobacco
    b. Yes, very difficult
    c. Yes, somewhat difficult
    d. No, not usually difficult
    e. No, not at all difficult

67. Do you think you will reduce tobacco use if the prices of tobacco products go up? (Select only one response)
    a. I have stopped using tobacco
    b. Yes, I will reduce the use of tobacco products
    c. Instead or reducing tobacco use, I will prefer to shift to cheaper tobacco products available
    d. No, price will not affect my tobacco use

## Media promotion of tobacco use

68. During the last 1-year, when you watched TV, video or movies, how often did you see actors smoking? (Select only one response)
    a. I did not watch TV, video or movies in the last 1 year
    b. Sometimes I have seen actors smoking
    c. A lot of the times I have seen actors smoking
    d. I have never seen them smoking

69. During the past 30 days when you watched TV (sport events, serials, other programmes), how often did you see cigarette advertisements or cigarette brand names? (Select only one response)
    a. I did not watch TV in the past 30 days
    b. Sometimes I have seen cigarette advertisements/brand names
    c. A lot of the times I have seen cigarette advertisement/brand names
    d. I have never seen cigarette advertisement/brand names

70. During the past 30 days when you watched TV (sport events, serials, other programmes), how often did you see bidi advertisements/brand names? (Select only one response)
    a. I did not watch TV in the past 30 days
    b. Sometimes I have seen bidi advertisements/brand names

c. A lot of the times I have seen bidi advertisements/brand names
d. I have never seen bidi advertisements/brand names

71. During the past 30 days when you watched TV (sport events, serials, other programmes), how often did you see gutka/khaini/pan-masala advertisements/brand names? (Select only one response)
    a. I did not watch TV in the past 30 days
    b. Sometimes I have seen such advertisements/brand names
    c. A lot of the times I have seen such advertisements/brand names
    d. I have never seen such advertisements/brand names

**Help/advise on tobacco use**

72. Has anyone in your family discussed the harmful effects of tobacco with you? (Select as many responses as applicable)
    a. Father    b. Mother    c. Brother
    d. Sister    e. Others in family (please specify)____________
    f. None of them

73. Have you ever received any assistance or advice to help you stop using tobacco? (Select as many responses as applicable)
    a. Yes, I received help/advise from a health professional (doctor, nurse etc.)
    b. Yes, I received help/advise from a family member
    c. Yes, I received help/advise from a friend
    d. Yes, I received help/advise during some programme by programme person
    e. Yes, I received help/advise from a teacher
    f. Yes, I received help/advise from TV/radio
    g. Yes, I received help/advise from news paper/magazine/books etc.
    h. No, I did not receive any help/advise
    i. Yes, I received help/advise from others (please specify)______

74. During the past 30 days (one month) how many anti-tobacco media messages have you seen (e.g. on TV, radio, hoardings, posters, newspapers, magazines etc. Select only one response)?
    a. A lot    b. A few    c. None

75. During the last year, were you taught in any of your classes about the dangers of smoking or chewing tobacco? (Select only one response)
    a. Yes    b. No    c. I am not sure

## Access to tobacco

76. During the past 30 days (one month), how did you usually get your bidis, cigarettes or any other tobacco products? (Select as many responses as applicable)
    a. I did not use any tobacco products in the past 30 days
    b. I purchased them from a store/shop/street vendor
    c. It was available at home
    d. I gave money to someone else to buy them for me
    e. I borrowed them from someone else
    f. I picked it from somewhere
    g. An older person gave them to me
    h. I got them some other way (please specify) ________________
    ________________________________________________________

77. How do you usually buy your cigarettes or bidis? (Select only one response)
    a. I do not smoke cigarettes or bidis
    b. I smoke them but do not buy them for me
    c. I buy loose cigarettes or bidis
    d. I buy them in a pack or bundle

78. During the past 30 days (one month) did anyone ever refuse to sell you cigarettes or bidis because of your age? (Select only one response)
    a. I did not try to buy cigarettes or bidis in the past 30 days
    b. Yes, I was sometimes refused sale of cigarettes or bidis because of my age
    c. Yes, I was always refused sale of cigarettes or bidis because of my age
    d. No, I was never refused sale of cigarettes or bidis because of my age

79. During the past 30 days (one month) did anyone ever refuse to sell you chewing tobacco products (gutka, khaini, pan-masala, pan with tobacco etc.) because of your age? (Select only one response)
    a. I did not try to buy chewing tobacco products in the past 30 days
    b. Yes, I was sometimes refused sale of chewing tobacco products because of my age
    c. Yes, I was always refused sale of chewing tobacco products because of my age
    d. No, I was never refused sale of chewing tobacco products because of my age

80. Is there any rule at your college that prohibits tobacco use and imposes punishments on those who use tobacco on campus? (Select only one response)
    a. I don't know
    b. There are no such rules or punishments
    c. There is a rule that prohibits tobacco use at my PUC usually those who use tobacco are not punished
    d. There is a rule that prohibits tobacco use at my PUC and those who use tobacco often get punished.

## Suggestions

81. According to you, what should be done to reduce tobacco use among youth (particularly pre-university students)? (Select only one answer)
    a. There is no need to do anything to reduce tobacco use among youth
    b. I feel that following things need to be done to reduce tobacco use among youth

    __________________________________________________

    __________________________________________________

    __________________________________________________

**Thank you very much!**

We highly appreciate the time and efforts you put in filling this questionnaire.

For any queries or further information on this research project or in general about tobacco use, please feel free to contact Dr. Upendra Bhojani by calling 9342349121 or by an email to drupendrabhojani@gmail.com
